# 4,6,10,12,16,18,22,24-Octa-*O*-methyl-2,8,14,20-tetra­pentylresorcin[4]arene

**DOI:** 10.1107/S1600536811034003

**Published:** 2011-09-14

**Authors:** Pramod B. Pansuriya, Holger B. Friedrich, Glenn E. M. Maguire

**Affiliations:** a School of Chemistry, University of KwaZulu-Natal, Durban 4000, South Africa

## Abstract

The complete molecule of the title compound, C_56_H_80_O_8_, is generated by a crystallographic inversion centre. The dihedral angle between the aromatic ring and the unique half of the molecule is 81.52 (16)°. There are no π–π inter­actions in the crystal structure.

## Related literature

For literature related to applications of resorcin[4]arenes, see: Gibson & Rebek (2002 ▶); Kim *et al.* (2005 ▶); Liu *et al.* (2010 ▶); D’Acquarica *et al.* (2011 ▶). For related structures, see: Botta *et al.* (2007 ▶); Iwanek (1998 ▶); Davis *et al.* (2001 ▶); Gerkensmeier *et al.* (2001 ▶); Moore & Matthews (2009 ▶).
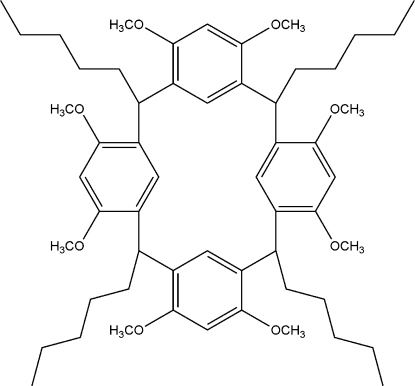


## Experimental

### 

#### Crystal data

C_56_H_80_O_8_
*M* *_r_* = 881.20
Triclinic, 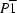

*a* = 8.2084 (2) Å
*b* = 14.1270 (2) Å
*c* = 23.1489 (4) Å
α = 98.929 (1)°
β = 97.914 (1)°
γ = 103.581 (1)°
*V* = 2534.93 (8) Å^3^
*Z* = 2
Mo *K*α radiation
μ = 0.08 mm^−1^
*T* = 173 K
0.44 × 0.39 × 0.13 mm

#### Data collection

Bruker APEXII CCD diffractometer
42741 measured reflections
12226 independent reflections
7816 reflections with *I* > 2σ(*I*)
*R*_int_ = 0.046

#### Refinement

*R*[*F* ^2^ > 2σ(*F* ^2^)] = 0.051
*wR*(*F* ^2^) = 0.148
*S* = 0.99
12226 reflections
589 parameters
H-atom parameters constrained
Δρ_max_ = 0.39 e Å^−3^
Δρ_min_ = −0.26 e Å^−3^

### 

Data collection: *APEX2* (Bruker, 2006 ▶); cell refinement: *SAINT* (Bruker, 2006 ▶); data reduction: *SAINT*; program(s) used to solve structure: *SHELXS97* (Sheldrick, 2008 ▶); program(s) used to refine structure: *SHELXL97* (Sheldrick, 2008 ▶); molecular graphics: *OLEX2* (Dolomanov *et al.*, 2009 ▶); software used to prepare material for publication: *SHELXTL* (Sheldrick, 2008 ▶).

## Supplementary Material

Crystal structure: contains datablock(s) I, global. DOI: 10.1107/S1600536811034003/hg5082sup1.cif

Structure factors: contains datablock(s) I. DOI: 10.1107/S1600536811034003/hg5082Isup2.hkl

Additional supplementary materials:  crystallographic information; 3D view; checkCIF report

## Figures and Tables

**Fig. 1.**
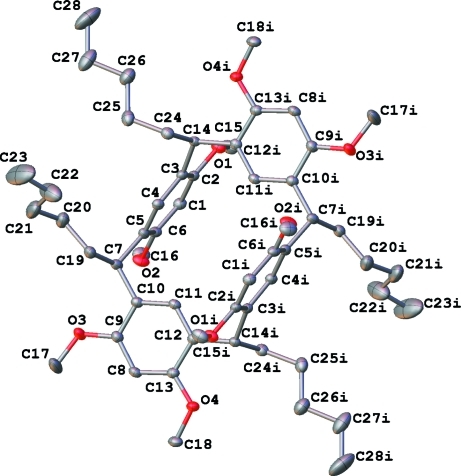
The molecular structure of the title compound with atomic numbering scheme. The H atoms have been omitted clarity. Displacement ellipsoids are drawn at 40% probability, symmetry code (i): 1 - x, -y, -z.

**Fig. 2.**
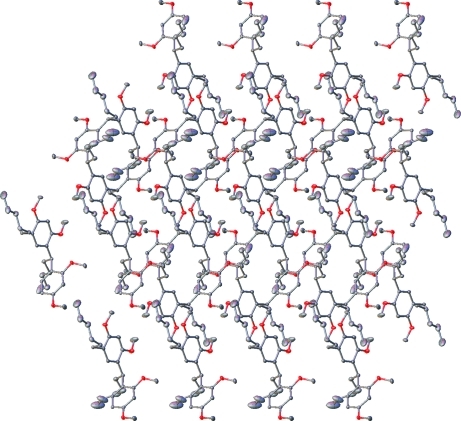
The packing of the title compound along the [110] axis. All H atoms have been omitted for clarity.

## supplementary crystallographic information

### Comment

Resorcin[4]arenes are easily prepared macromolecules that are used for a range of applications in supramolecular chemistry such as catalysis (Gibson & Rebek, 2002), self-assembled nanoparticles (Kim *et al.*, 2005), molecular recognition (Liu *et al.*, 2010) and noncompetitive inhibitors for *R*-chymotrypsin (D'Acquarica *et al.*, 2011).

The different conformers of resorcin[4]arenes that can occur in solution have been extensively reported (Moore & Matthews, 2009). Their topology has been classified into four diffrent structures *i.e. rccc* (cone/crown), rctt (chair), *rctc* (diamond), *rccc* (boat). The most commonly reported single-crystal X-ray structures have been the crown and boat isomers (Davis *et al.*, 2001; Gerkensmeier *et al.*, 2001). In the title compound, a pair of aromatic rings are almost coplanar (C8—C9—C11—C12—C13), whereas the others are orthogonal at an angle of 86.91° from the plane facing the side-chains. This creates an *rctt* conformation (Fig 1). Botta *et al.* have described the only other *rctt* octamethoxy-resorcin[4]arene structure thus far (Botta *et al.*, 2007) which had ethyl ester side chains. Iwanek have reported the only *rctc* octamethoxy resorcinarene (Iwanek, 1998) with isopropyl side chains. There are no π–π interactions in the crystal matrix (Fig. 2).

### Experimental

1,3-Dimethoxybenzene (1.054 g, 7.63 mmol) and hexanal (0.764 g, 7.63 mmol) were added to diethyl ether (20 ml) followed by addition of thionyl chloride (0.907 g, 7.63 mmol). The solution was stirred at room temperature for 24 h. Subsequently, methanol (40 ml) was added, and the mixture was stirred for 1 h. The resulting precipitate was collected by filtration and washed with methanol. The yield was 0.65 g (36%). m.p. = 516–519 K.

Crystals suitable for single-crystal X-ray diffraction were grown in methanol: methylene chloride (1:2) at room temperature.

### Refinement

H atoms were first located in the difference map then positioned geometrically and allowed to ride on their respective parent atoms with C—H = 0.95 Å and *U_i_*_so_(H) = 1.2*U*_eq_(C) for aromatic and C—H = 0.99 Å and *U_i_*_so_(H) = 1.2*U*_eq_(C) for the aliphatic chain.

### Crystal data

**Table:**

C_56_H_80_O_8_	*Z* = 2
*M**_r_* = 881.20	*F*(000) = 960
Triclinic, *P*1	*D*_x_ = 1.154 Mg m^−^^3^
Hall symbol: -P 1	Mo *K*α radiation, λ = 0.71073 Å
*a* = 8.2084 (2) Å	Cell parameters from 9018 reflections
*b* = 14.1270 (2) Å	θ = 2.6–28.0°
*c* = 23.1489 (4) Å	µ = 0.08 mm^−^^1^
α = 98.929 (1)°	*T* = 173 K
β = 97.914 (1)°	Plate, colourless
γ = 103.581 (1)°	0.44 × 0.39 × 0.13 mm
*V* = 2534.93 (8) Å^3^	

### Data collection

**Table:**

Bruker APEXII CCD diffractometer	7816 reflections with *I* > 2σ(*I*)
Radiation source: fine-focus sealed tube	*R*_int_ = 0.046
graphite	θ_max_ = 28.0°, θ_min_ = 0.9°
φ and ω scans	*h* = −10→10
42741 measured reflections	*k* = −18→18
12226 independent reflections	*l* = −30→30

### Refinement

**Table:**

Refinement on *F*^2^	Primary atom site location: structure-invariant direct methods
Least-squares matrix: full	Secondary atom site location: difference Fourier map
*R*[*F*^2^ > 2σ(*F*^2^)] = 0.051	Hydrogen site location: inferred from neighbouring sites
*wR*(*F*^2^) = 0.148	H-atom parameters constrained
*S* = 0.99	*w* = 1/[σ^2^(*F*_o_^2^) + (0.0817*P*)^2^] where *P* = (*F*_o_^2^ + 2*F*_c_^2^)/3
12226 reflections	(Δ/σ)_max_ = 0.001
589 parameters	Δρ_max_ = 0.39 e Å^−^^3^
0 restraints	Δρ_min_ = −0.26 e Å^−^^3^

### Special details

**Table:**

Geometry. All e.s.d.'s (except the e.s.d. in the dihedral angle between two l.s. planes) are estimated using the full covariance matrix. The cell e.s.d.'s are taken into account individually in the estimation of e.s.d.'s in distances, angles and torsion angles; correlations between e.s.d.'s in cell parameters are only used when they are defined by crystal symmetry. An approximate (isotropic) treatment of cell e.s.d.'s is used for estimating e.s.d.'s involving l.s. planes.
Refinement. Refinement of *F*^2^ against ALL reflections. The weighted *R*-factor *wR* and goodness of fit *S* are based on *F*^2^, conventional *R*-factors *R* are based on *F*, with *F* set to zero for negative *F*^2^. The threshold expression of *F*^2^ > σ(*F*^2^) is used only for calculating *R*-factors(gt) *etc*. and is not relevant to the choice of reflections for refinement. *R*-factors based on *F*^2^ are statistically about twice as large as those based on *F*, and *R*-factors based on ALL data will be even larger.

### Fractional atomic coordinates and isotropic or equivalent isotropic displacement parameters (Å^2^)

**Table:**

	*x*	*y*	*z*	*U*_iso_*/*U*_eq_	
C1	0.57192 (19)	0.04899 (10)	−0.06674 (6)	0.0238 (3)	
H1	0.4525	0.0246	−0.0692	0.029*	
C2	0.65461 (18)	0.00564 (10)	−0.10787 (6)	0.0207 (3)	
C3	0.82972 (18)	0.04165 (10)	−0.10628 (6)	0.0195 (3)	
C4	0.91774 (18)	0.11977 (10)	−0.05978 (6)	0.0208 (3)	
H4	1.0373	0.1438	−0.0571	0.025*	
C5	0.84136 (19)	0.16501 (10)	−0.01687 (6)	0.0219 (3)	
C6	0.66495 (19)	0.12805 (10)	−0.02208 (6)	0.0235 (3)	
C7	0.9370 (2)	0.25271 (10)	0.03240 (6)	0.0239 (3)	
H7	0.8554	0.2941	0.0391	0.029*	
C8	1.03311 (19)	0.26193 (10)	0.19982 (6)	0.0246 (3)	
H8	1.0392	0.3073	0.2354	0.029*	
C9	0.99799 (19)	0.28811 (10)	0.14464 (6)	0.0244 (3)	
C10	0.98417 (18)	0.22153 (10)	0.09158 (6)	0.0221 (3)	
C11	1.01599 (18)	0.13015 (10)	0.09603 (6)	0.0209 (3)	
H11	1.0101	0.0848	0.0604	0.025*	
C12	1.05618 (18)	0.10183 (10)	0.15035 (6)	0.0200 (3)	
C13	1.05902 (18)	0.16881 (10)	0.20215 (6)	0.0221 (3)	
C14	1.08814 (17)	0.00129 (10)	0.15609 (6)	0.0189 (3)	
H14	1.1731	0.0135	0.1936	0.023*	
C15	0.3894 (2)	−0.10994 (12)	−0.15630 (7)	0.0338 (4)	
H15A	0.3671	−0.1293	−0.1187	0.051*	
H15B	0.3444	−0.1677	−0.1888	0.051*	
H15C	0.3336	−0.0577	−0.1637	0.051*	
C16	0.4127 (2)	0.16205 (13)	0.00749 (8)	0.0373 (4)	
H16A	0.3828	0.1829	−0.0301	0.056*	
H16B	0.3788	0.2029	0.0396	0.056*	
H16C	0.3531	0.0921	0.0043	0.056*	
C17	1.0271 (3)	0.45754 (13)	0.18855 (9)	0.0643 (7)	
H17A	0.9504	0.4463	0.2172	0.096*	
H17B	1.0256	0.5204	0.1758	0.096*	
H17C	1.1432	0.4604	0.2073	0.096*	
C18	1.0755 (2)	0.19585 (11)	0.30801 (7)	0.0300 (4)	
H18A	1.1562	0.2614	0.3144	0.045*	
H18B	1.1003	0.1636	0.3415	0.045*	
H18C	0.9591	0.2034	0.3049	0.045*	
C19	1.0918 (2)	0.32067 (11)	0.01602 (7)	0.0289 (4)	
H19A	1.1528	0.3718	0.0515	0.035*	
H19B	1.1707	0.2808	0.0045	0.035*	
C20	1.0443 (3)	0.37200 (13)	−0.03473 (8)	0.0459 (5)	
H20A	0.9915	0.3207	−0.0710	0.055*	
H20B	0.9576	0.4069	−0.0245	0.055*	
C21	1.1938 (3)	0.44644 (15)	−0.04869 (10)	0.0658 (7)	
H21A	1.1493	0.4820	−0.0783	0.079*	
H21B	1.2496	0.4961	−0.0120	0.079*	
C22	1.3244 (4)	0.40150 (18)	−0.07211 (12)	0.0925 (10)	
H22A	1.2664	0.3424	−0.1035	0.111*	
H22B	1.3876	0.3791	−0.0396	0.111*	
C23	1.4507 (4)	0.4738 (2)	−0.09782 (13)	0.1321 (16)	
H23A	1.3920	0.4874	−0.1342	0.198*	
H23B	1.5432	0.4444	−0.1071	0.198*	
H23C	1.4979	0.5359	−0.0687	0.198*	
C24	0.92534 (19)	−0.07005 (10)	0.16585 (7)	0.0255 (3)	
H24A	0.8710	−0.0325	0.1937	0.031*	
H24B	0.8449	−0.0933	0.1274	0.031*	
C25	0.9520 (2)	−0.16053 (11)	0.19023 (8)	0.0348 (4)	
H25A	0.8401	−0.2095	0.1847	0.042*	
H25B	1.0242	−0.1916	0.1666	0.042*	
C26	1.0343 (3)	−0.13830 (14)	0.25552 (9)	0.0462 (5)	
H26A	0.9687	−0.1010	0.2787	0.055*	
H26B	1.1510	−0.0948	0.2603	0.055*	
C27	1.0449 (3)	−0.22990 (16)	0.28167 (10)	0.0611 (6)	
H27A	0.9287	−0.2744	0.2760	0.073*	
H27B	1.1136	−0.2662	0.2595	0.073*	
C28	1.1227 (4)	−0.2061 (2)	0.34712 (12)	0.0945 (10)	
H28A	1.2352	−0.1591	0.3535	0.142*	
H28B	1.1344	−0.2672	0.3603	0.142*	
H28C	1.0488	−0.1766	0.3700	0.142*	
C29	0.92260 (19)	0.44372 (10)	0.56863 (6)	0.0225 (3)	
H29	1.0429	0.4674	0.5734	0.027*	
C30	0.83704 (18)	0.47882 (9)	0.61160 (6)	0.0196 (3)	
C31	0.66025 (17)	0.44376 (9)	0.60632 (6)	0.0172 (3)	
C32	0.57479 (18)	0.37462 (9)	0.55514 (6)	0.0172 (3)	
H32	0.4544	0.3515	0.5501	0.021*	
C33	0.65478 (18)	0.33708 (9)	0.51066 (6)	0.0178 (3)	
C34	0.83149 (18)	0.37388 (10)	0.51865 (6)	0.0203 (3)	
C35	0.56261 (18)	0.25566 (9)	0.45731 (6)	0.0187 (3)	
H35	0.6422	0.2131	0.4502	0.022*	
C36	0.49061 (18)	0.26418 (10)	0.29213 (6)	0.0210 (3)	
H36	0.4891	0.2221	0.2557	0.025*	
C37	0.52010 (18)	0.23300 (10)	0.34572 (6)	0.0200 (3)	
C38	0.52755 (18)	0.29423 (10)	0.40005 (6)	0.0184 (3)	
C39	0.49580 (17)	0.38639 (9)	0.39833 (6)	0.0184 (3)	
H39	0.4980	0.4285	0.4348	0.022*	
C40	0.46084 (17)	0.41983 (9)	0.34553 (6)	0.0173 (3)	
C41	0.46333 (18)	0.35759 (10)	0.29239 (6)	0.0191 (3)	
C42	0.42842 (17)	0.52173 (9)	0.34370 (6)	0.0175 (3)	
H42	0.3481	0.5141	0.3056	0.021*	
C43	1.0998 (2)	0.58625 (13)	0.66741 (8)	0.0365 (4)	
H43A	1.1532	0.5312	0.6683	0.055*	
H43B	1.1440	0.6354	0.7044	0.055*	
H43C	1.1260	0.6174	0.6336	0.055*	
C44	1.0860 (2)	0.34697 (12)	0.48723 (7)	0.0324 (4)	
H44A	1.1474	0.4175	0.4968	0.049*	
H44B	1.1223	0.3140	0.4527	0.049*	
H44C	1.1112	0.3161	0.5213	0.049*	
C45	0.5143 (3)	0.07023 (12)	0.29651 (8)	0.0532 (6)	
H45A	0.4027	0.0656	0.2731	0.080*	
H45B	0.5165	0.0054	0.3062	0.080*	
H45C	0.6034	0.0905	0.2734	0.080*	
C46	0.4490 (2)	0.33674 (11)	0.18674 (6)	0.0304 (4)	
H46A	0.5665	0.3314	0.1885	0.046*	
H46B	0.4189	0.3690	0.1537	0.046*	
H46C	0.3715	0.2702	0.1805	0.046*	
C47	0.39980 (19)	0.18682 (10)	0.46791 (6)	0.0225 (3)	
H47A	0.3190	0.2268	0.4771	0.027*	
H47B	0.3461	0.1378	0.4308	0.027*	
C48	0.4288 (2)	0.13113 (10)	0.51825 (7)	0.0256 (3)	
H48A	0.5097	0.0912	0.5093	0.031*	
H48B	0.4817	0.1799	0.5555	0.031*	
C49	0.2662 (2)	0.06318 (13)	0.52775 (8)	0.0374 (4)	
H49A	0.1814	0.1019	0.5330	0.045*	
H49B	0.2189	0.0106	0.4918	0.045*	
C50	0.2925 (2)	0.01513 (13)	0.58115 (8)	0.0406 (4)	
H50A	0.3348	0.0677	0.6174	0.049*	
H50B	0.3811	−0.0211	0.5767	0.049*	
C51	0.1312 (3)	−0.0566 (2)	0.58920 (13)	0.0899 (10)	
H51A	0.0454	−0.0204	0.5964	0.135*	
H51B	0.1572	−0.0875	0.6232	0.135*	
H51C	0.0871	−0.1081	0.5532	0.135*	
C52	0.59415 (19)	0.59643 (10)	0.33991 (6)	0.0221 (3)	
H52A	0.6719	0.6113	0.3788	0.027*	
H52B	0.6497	0.5646	0.3099	0.027*	
C53	0.5731 (2)	0.69418 (10)	0.32391 (7)	0.0297 (4)	
H53A	0.4973	0.7195	0.3486	0.036*	
H53B	0.6857	0.7434	0.3340	0.036*	
C54	0.5003 (3)	0.68609 (13)	0.25889 (8)	0.0410 (4)	
H54A	0.3838	0.6412	0.2497	0.049*	
H54B	0.5708	0.6556	0.2341	0.049*	
C55	0.4927 (3)	0.78484 (15)	0.24201 (10)	0.0575 (6)	
H55A	0.4229	0.8156	0.2669	0.069*	
H55B	0.6093	0.8296	0.2509	0.069*	
C56	0.4189 (4)	0.7761 (2)	0.17737 (12)	0.0945 (10)	
H56A	0.4914	0.7498	0.1523	0.142*	
H56B	0.4135	0.8418	0.1698	0.142*	
H56C	0.3039	0.7312	0.1680	0.142*	
O1	0.56951 (13)	−0.07364 (7)	−0.15298 (4)	0.0257 (2)	
O2	0.59201 (14)	0.17402 (8)	0.02027 (5)	0.0325 (3)	
O3	0.97265 (16)	0.37925 (8)	0.13885 (5)	0.0350 (3)	
O4	1.09114 (14)	0.13645 (7)	0.25449 (4)	0.0283 (3)	
O5	0.91909 (13)	0.54939 (7)	0.66135 (4)	0.0261 (2)	
O6	0.90743 (13)	0.33729 (8)	0.47391 (4)	0.0278 (2)	
O7	0.54300 (15)	0.14038 (7)	0.34895 (4)	0.0301 (3)	
O8	0.43475 (14)	0.39443 (7)	0.24128 (4)	0.0263 (2)	

### Atomic displacement parameters (Å^2^)

**Table:**

	*U*^11^	*U*^22^	*U*^33^	*U*^12^	*U*^13^	*U*^23^
C1	0.0197 (8)	0.0303 (8)	0.0234 (8)	0.0085 (6)	0.0057 (6)	0.0063 (6)
C2	0.0215 (8)	0.0224 (7)	0.0174 (7)	0.0059 (6)	0.0010 (6)	0.0041 (5)
C3	0.0198 (8)	0.0232 (7)	0.0171 (7)	0.0085 (6)	0.0026 (6)	0.0052 (5)
C4	0.0185 (7)	0.0250 (7)	0.0198 (7)	0.0084 (6)	0.0018 (6)	0.0042 (6)
C5	0.0245 (8)	0.0223 (7)	0.0195 (7)	0.0098 (6)	0.0004 (6)	0.0033 (6)
C6	0.0262 (8)	0.0294 (8)	0.0187 (7)	0.0139 (7)	0.0060 (6)	0.0043 (6)
C7	0.0280 (8)	0.0252 (7)	0.0191 (7)	0.0128 (6)	0.0003 (6)	0.0002 (6)
C8	0.0259 (8)	0.0253 (7)	0.0193 (7)	0.0056 (6)	0.0032 (6)	−0.0029 (6)
C9	0.0249 (8)	0.0238 (7)	0.0234 (8)	0.0076 (6)	0.0032 (6)	0.0007 (6)
C10	0.0197 (8)	0.0258 (7)	0.0199 (7)	0.0066 (6)	0.0024 (6)	0.0021 (6)
C11	0.0183 (7)	0.0239 (7)	0.0185 (7)	0.0052 (6)	0.0032 (6)	−0.0009 (6)
C12	0.0151 (7)	0.0232 (7)	0.0203 (7)	0.0037 (6)	0.0031 (6)	0.0019 (6)
C13	0.0195 (8)	0.0266 (7)	0.0185 (7)	0.0037 (6)	0.0030 (6)	0.0033 (6)
C14	0.0171 (7)	0.0217 (7)	0.0164 (7)	0.0050 (6)	0.0013 (5)	0.0011 (5)
C15	0.0189 (8)	0.0413 (9)	0.0336 (9)	0.0016 (7)	0.0038 (7)	−0.0052 (7)
C16	0.0304 (10)	0.0522 (10)	0.0357 (10)	0.0234 (8)	0.0100 (8)	0.0049 (8)
C17	0.116 (2)	0.0300 (10)	0.0376 (11)	0.0288 (12)	−0.0175 (12)	−0.0077 (8)
C18	0.0345 (9)	0.0345 (8)	0.0193 (8)	0.0061 (7)	0.0075 (7)	0.0019 (6)
C19	0.0347 (9)	0.0233 (7)	0.0249 (8)	0.0056 (7)	0.0008 (7)	0.0010 (6)
C20	0.0613 (13)	0.0311 (9)	0.0397 (11)	0.0055 (9)	−0.0024 (9)	0.0103 (8)
C21	0.0915 (19)	0.0416 (11)	0.0523 (13)	−0.0068 (12)	−0.0001 (13)	0.0225 (10)
C22	0.115 (2)	0.0700 (16)	0.0653 (17)	−0.0333 (16)	0.0468 (17)	−0.0061 (13)
C23	0.139 (3)	0.136 (3)	0.072 (2)	−0.070 (2)	0.023 (2)	0.0325 (19)
C24	0.0224 (8)	0.0280 (8)	0.0258 (8)	0.0058 (6)	0.0074 (6)	0.0027 (6)
C25	0.0353 (10)	0.0287 (8)	0.0433 (10)	0.0063 (7)	0.0173 (8)	0.0094 (7)
C26	0.0498 (12)	0.0475 (11)	0.0482 (12)	0.0150 (9)	0.0130 (10)	0.0223 (9)
C27	0.0573 (14)	0.0705 (14)	0.0784 (17)	0.0280 (12)	0.0308 (13)	0.0490 (13)
C28	0.098 (2)	0.136 (3)	0.084 (2)	0.052 (2)	0.0280 (17)	0.079 (2)
C29	0.0169 (7)	0.0254 (7)	0.0250 (8)	0.0057 (6)	0.0044 (6)	0.0037 (6)
C30	0.0202 (8)	0.0185 (7)	0.0190 (7)	0.0054 (6)	0.0014 (6)	0.0019 (5)
C31	0.0193 (7)	0.0173 (6)	0.0176 (7)	0.0077 (6)	0.0039 (5)	0.0060 (5)
C32	0.0155 (7)	0.0178 (6)	0.0192 (7)	0.0054 (5)	0.0019 (5)	0.0057 (5)
C33	0.0211 (7)	0.0168 (6)	0.0174 (7)	0.0081 (6)	0.0020 (6)	0.0049 (5)
C34	0.0231 (8)	0.0219 (7)	0.0192 (7)	0.0102 (6)	0.0070 (6)	0.0047 (6)
C35	0.0237 (8)	0.0184 (6)	0.0147 (7)	0.0090 (6)	0.0009 (6)	0.0025 (5)
C36	0.0241 (8)	0.0205 (7)	0.0172 (7)	0.0058 (6)	0.0036 (6)	0.0007 (5)
C37	0.0223 (8)	0.0174 (6)	0.0205 (7)	0.0067 (6)	0.0030 (6)	0.0028 (5)
C38	0.0176 (7)	0.0202 (7)	0.0170 (7)	0.0051 (6)	0.0022 (5)	0.0031 (5)
C39	0.0188 (7)	0.0192 (6)	0.0162 (7)	0.0045 (6)	0.0028 (6)	0.0022 (5)
C40	0.0158 (7)	0.0168 (6)	0.0196 (7)	0.0044 (5)	0.0040 (5)	0.0037 (5)
C41	0.0207 (7)	0.0200 (7)	0.0160 (7)	0.0038 (6)	0.0023 (6)	0.0048 (5)
C42	0.0193 (7)	0.0174 (6)	0.0157 (7)	0.0061 (6)	0.0021 (5)	0.0021 (5)
C43	0.0180 (8)	0.0458 (10)	0.0355 (9)	0.0021 (7)	0.0019 (7)	−0.0108 (8)
C44	0.0256 (9)	0.0426 (9)	0.0337 (9)	0.0185 (7)	0.0094 (7)	0.0039 (7)
C45	0.0998 (18)	0.0261 (9)	0.0289 (10)	0.0269 (10)	−0.0118 (10)	−0.0045 (7)
C46	0.0438 (10)	0.0317 (8)	0.0169 (7)	0.0108 (7)	0.0074 (7)	0.0048 (6)
C47	0.0263 (8)	0.0193 (7)	0.0213 (7)	0.0061 (6)	0.0014 (6)	0.0039 (6)
C48	0.0297 (9)	0.0228 (7)	0.0249 (8)	0.0066 (6)	0.0035 (6)	0.0079 (6)
C49	0.0338 (10)	0.0387 (9)	0.0422 (10)	0.0058 (8)	0.0060 (8)	0.0218 (8)
C50	0.0422 (11)	0.0429 (10)	0.0439 (11)	0.0115 (8)	0.0125 (9)	0.0251 (8)
C51	0.0470 (14)	0.123 (2)	0.126 (2)	0.0163 (14)	0.0252 (15)	0.104 (2)
C52	0.0230 (8)	0.0219 (7)	0.0230 (7)	0.0063 (6)	0.0076 (6)	0.0053 (6)
C53	0.0356 (10)	0.0213 (7)	0.0358 (9)	0.0069 (7)	0.0156 (7)	0.0089 (7)
C54	0.0529 (12)	0.0415 (10)	0.0394 (10)	0.0214 (9)	0.0156 (9)	0.0200 (8)
C55	0.0609 (14)	0.0596 (12)	0.0794 (16)	0.0330 (11)	0.0356 (12)	0.0489 (12)
C56	0.110 (2)	0.136 (2)	0.092 (2)	0.079 (2)	0.0461 (18)	0.090 (2)
O1	0.0166 (5)	0.0309 (5)	0.0248 (6)	0.0030 (4)	0.0032 (4)	−0.0031 (4)
O2	0.0275 (6)	0.0447 (7)	0.0265 (6)	0.0164 (5)	0.0079 (5)	−0.0032 (5)
O3	0.0528 (8)	0.0250 (5)	0.0251 (6)	0.0174 (5)	−0.0037 (5)	−0.0030 (4)
O4	0.0382 (7)	0.0284 (5)	0.0171 (5)	0.0084 (5)	0.0040 (5)	0.0025 (4)
O5	0.0169 (5)	0.0300 (5)	0.0247 (6)	0.0017 (4)	0.0022 (4)	−0.0063 (4)
O6	0.0225 (6)	0.0368 (6)	0.0244 (6)	0.0115 (5)	0.0074 (4)	−0.0019 (5)
O7	0.0506 (8)	0.0198 (5)	0.0205 (5)	0.0172 (5)	−0.0007 (5)	−0.0005 (4)
O8	0.0404 (7)	0.0240 (5)	0.0161 (5)	0.0109 (5)	0.0049 (5)	0.0052 (4)

### Geometric parameters (Å, °)

**Table:**

C1—C6	1.387 (2)	C29—C30	1.3896 (19)
C1—C2	1.3908 (19)	C29—C34	1.3900 (19)
C1—H1	0.9500	C29—H29	0.9500
C2—O1	1.3790 (16)	C30—O5	1.3743 (16)
C2—C3	1.4009 (19)	C30—C31	1.4004 (19)
C3—C4	1.3933 (19)	C31—C32	1.3928 (18)
C3—C14^i^	1.5299 (18)	C31—C42^ii^	1.5263 (17)
C4—C5	1.3968 (19)	C32—C33	1.3972 (18)
C4—H4	0.9500	C32—H32	0.9500
C5—C6	1.400 (2)	C33—C34	1.3967 (19)
C5—C7	1.5200 (19)	C33—C35	1.5211 (18)
C6—O2	1.3740 (16)	C34—O6	1.3752 (15)
C7—C10	1.530 (2)	C35—C38	1.5262 (18)
C7—C19	1.534 (2)	C35—C47	1.5317 (19)
C7—H7	1.0000	C35—H35	1.0000
C8—C13	1.389 (2)	C36—C41	1.3886 (18)
C8—C9	1.395 (2)	C36—C37	1.3887 (19)
C8—H8	0.9500	C36—H36	0.9500
C9—O3	1.3750 (17)	C37—O7	1.3765 (15)
C9—C10	1.3996 (19)	C37—C38	1.3965 (18)
C10—C11	1.3919 (19)	C38—C39	1.3919 (18)
C11—C12	1.3980 (19)	C39—C40	1.3947 (19)
C11—H11	0.9500	C39—H39	0.9500
C12—C13	1.4014 (18)	C40—C41	1.4023 (18)
C12—C14	1.5257 (18)	C40—C42	1.5300 (17)
C13—O4	1.3736 (17)	C41—O8	1.3777 (16)
C14—C3^i^	1.5299 (18)	C42—C31^ii^	1.5263 (17)
C14—C24	1.5407 (19)	C42—C52	1.5360 (18)
C14—H14	1.0000	C42—H42	1.0000
C15—O1	1.4335 (17)	C43—O5	1.4314 (18)
C15—H15A	0.9800	C43—H43A	0.9800
C15—H15B	0.9800	C43—H43B	0.9800
C15—H15C	0.9800	C43—H43C	0.9800
C16—O2	1.4249 (19)	C44—O6	1.4252 (18)
C16—H16A	0.9800	C44—H44A	0.9800
C16—H16B	0.9800	C44—H44B	0.9800
C16—H16C	0.9800	C44—H44C	0.9800
C17—O3	1.4064 (19)	C45—O7	1.3961 (18)
C17—H17A	0.9800	C45—H45A	0.9800
C17—H17B	0.9800	C45—H45B	0.9800
C17—H17C	0.9800	C45—H45C	0.9800
C18—O4	1.4241 (17)	C46—O8	1.4277 (16)
C18—H18A	0.9800	C46—H46A	0.9800
C18—H18B	0.9800	C46—H46B	0.9800
C18—H18C	0.9800	C46—H46C	0.9800
C19—C20	1.526 (2)	C47—C48	1.5264 (19)
C19—H19A	0.9900	C47—H47A	0.9900
C19—H19B	0.9900	C47—H47B	0.9900
C20—C21	1.525 (3)	C48—C49	1.516 (2)
C20—H20A	0.9900	C48—H48A	0.9900
C20—H20B	0.9900	C48—H48B	0.9900
C21—C22	1.489 (4)	C49—C50	1.514 (2)
C21—H21A	0.9900	C49—H49A	0.9900
C21—H21B	0.9900	C49—H49B	0.9900
C22—C23	1.528 (3)	C50—C51	1.520 (3)
C22—H22A	0.9900	C50—H50A	0.9900
C22—H22B	0.9900	C50—H50B	0.9900
C23—H23A	0.9800	C51—H51A	0.9800
C23—H23B	0.9800	C51—H51B	0.9800
C23—H23C	0.9800	C51—H51C	0.9800
C24—C25	1.524 (2)	C52—C53	1.5254 (19)
C24—H24A	0.9900	C52—H52A	0.9900
C24—H24B	0.9900	C52—H52B	0.9900
C25—C26	1.520 (2)	C53—C54	1.517 (2)
C25—H25A	0.9900	C53—H53A	0.9900
C25—H25B	0.9900	C53—H53B	0.9900
C26—C27	1.526 (2)	C54—C55	1.519 (2)
C26—H26A	0.9900	C54—H54A	0.9900
C26—H26B	0.9900	C54—H54B	0.9900
C27—C28	1.514 (3)	C55—C56	1.510 (3)
C27—H27A	0.9900	C55—H55A	0.9900
C27—H27B	0.9900	C55—H55B	0.9900
C28—H28A	0.9800	C56—H56A	0.9800
C28—H28B	0.9800	C56—H56B	0.9800
C28—H28C	0.9800	C56—H56C	0.9800
			
C6—C1—C2	119.56 (14)	O5—C30—C31	116.04 (12)
C6—C1—H1	120.2	C29—C30—C31	121.34 (13)
C2—C1—H1	120.2	C32—C31—C30	116.62 (12)
O1—C2—C1	122.32 (13)	C32—C31—C42^ii^	123.23 (12)
O1—C2—C3	116.16 (12)	C30—C31—C42^ii^	120.10 (12)
C1—C2—C3	121.51 (13)	C31—C32—C33	124.22 (13)
C4—C3—C2	116.56 (12)	C31—C32—H32	117.9
C4—C3—C14^i^	123.66 (13)	C33—C32—H32	117.9
C2—C3—C14^i^	119.69 (12)	C34—C33—C32	116.61 (12)
C3—C4—C5	124.16 (14)	C34—C33—C35	119.34 (12)
C3—C4—H4	117.9	C32—C33—C35	123.95 (13)
C5—C4—H4	117.9	O6—C34—C29	122.85 (13)
C4—C5—C6	116.57 (13)	O6—C34—C33	115.71 (12)
C4—C5—C7	123.90 (13)	C29—C34—C33	121.44 (12)
C6—C5—C7	119.48 (12)	C33—C35—C38	113.56 (11)
O2—C6—C1	122.73 (13)	C33—C35—C47	114.02 (11)
O2—C6—C5	115.69 (13)	C38—C35—C47	110.54 (12)
C1—C6—C5	121.57 (13)	C33—C35—H35	106.0
C5—C7—C10	112.62 (12)	C38—C35—H35	106.0
C5—C7—C19	114.10 (12)	C47—C35—H35	106.0
C10—C7—C19	111.39 (12)	C41—C36—C37	119.24 (12)
C5—C7—H7	106.0	C41—C36—H36	120.4
C10—C7—H7	106.0	C37—C36—H36	120.4
C19—C7—H7	106.0	O7—C37—C36	122.64 (12)
C13—C8—C9	119.31 (13)	O7—C37—C38	115.69 (12)
C13—C8—H8	120.3	C36—C37—C38	121.67 (12)
C9—C8—H8	120.3	C39—C38—C37	117.28 (12)
O3—C9—C8	122.60 (13)	C39—C38—C35	123.77 (12)
O3—C9—C10	116.08 (13)	C37—C38—C35	118.91 (12)
C8—C9—C10	121.32 (13)	C38—C39—C40	123.09 (12)
C11—C10—C9	117.39 (13)	C38—C39—H39	118.5
C11—C10—C7	123.70 (12)	C40—C39—H39	118.5
C9—C10—C7	118.91 (12)	C39—C40—C41	117.36 (12)
C10—C11—C12	123.19 (13)	C39—C40—C42	123.08 (12)
C10—C11—H11	118.4	C41—C40—C42	119.50 (12)
C12—C11—H11	118.4	O8—C41—C36	122.85 (12)
C11—C12—C13	117.26 (13)	O8—C41—C40	115.90 (11)
C11—C12—C14	123.93 (12)	C36—C41—C40	121.24 (12)
C13—C12—C14	118.74 (12)	C31^ii^—C42—C40	112.82 (10)
O4—C13—C8	123.07 (12)	C31^ii^—C42—C52	114.20 (11)
O4—C13—C12	115.54 (12)	C40—C42—C52	109.85 (11)
C8—C13—C12	121.38 (13)	C31^ii^—C42—H42	106.5
C12—C14—C3^i^	113.62 (11)	C40—C42—H42	106.5
C12—C14—C24	110.26 (11)	C52—C42—H42	106.5
C3^i^—C14—C24	114.38 (11)	O5—C43—H43A	109.5
C12—C14—H14	105.9	O5—C43—H43B	109.5
C3^i^—C14—H14	105.9	H43A—C43—H43B	109.5
C24—C14—H14	105.9	O5—C43—H43C	109.5
O1—C15—H15A	109.5	H43A—C43—H43C	109.5
O1—C15—H15B	109.5	H43B—C43—H43C	109.5
H15A—C15—H15B	109.5	O6—C44—H44A	109.5
O1—C15—H15C	109.5	O6—C44—H44B	109.5
H15A—C15—H15C	109.5	H44A—C44—H44B	109.5
H15B—C15—H15C	109.5	O6—C44—H44C	109.5
O2—C16—H16A	109.5	H44A—C44—H44C	109.5
O2—C16—H16B	109.5	H44B—C44—H44C	109.5
H16A—C16—H16B	109.5	O7—C45—H45A	109.5
O2—C16—H16C	109.5	O7—C45—H45B	109.5
H16A—C16—H16C	109.5	H45A—C45—H45B	109.5
H16B—C16—H16C	109.5	O7—C45—H45C	109.5
O3—C17—H17A	109.5	H45A—C45—H45C	109.5
O3—C17—H17B	109.5	H45B—C45—H45C	109.5
H17A—C17—H17B	109.5	O8—C46—H46A	109.5
O3—C17—H17C	109.5	O8—C46—H46B	109.5
H17A—C17—H17C	109.5	H46A—C46—H46B	109.5
H17B—C17—H17C	109.5	O8—C46—H46C	109.5
O4—C18—H18A	109.5	H46A—C46—H46C	109.5
O4—C18—H18B	109.5	H46B—C46—H46C	109.5
H18A—C18—H18B	109.5	C48—C47—C35	114.04 (12)
O4—C18—H18C	109.5	C48—C47—H47A	108.7
H18A—C18—H18C	109.5	C35—C47—H47A	108.7
H18B—C18—H18C	109.5	C48—C47—H47B	108.7
C20—C19—C7	113.16 (14)	C35—C47—H47B	108.7
C20—C19—H19A	108.9	H47A—C47—H47B	107.6
C7—C19—H19A	108.9	C49—C48—C47	113.11 (13)
C20—C19—H19B	108.9	C49—C48—H48A	109.0
C7—C19—H19B	108.9	C47—C48—H48A	109.0
H19A—C19—H19B	107.8	C49—C48—H48B	109.0
C21—C20—C19	114.37 (17)	C47—C48—H48B	109.0
C21—C20—H20A	108.7	H48A—C48—H48B	107.8
C19—C20—H20A	108.7	C50—C49—C48	113.07 (14)
C21—C20—H20B	108.7	C50—C49—H49A	109.0
C19—C20—H20B	108.7	C48—C49—H49A	109.0
H20A—C20—H20B	107.6	C50—C49—H49B	109.0
C22—C21—C20	114.38 (17)	C48—C49—H49B	109.0
C22—C21—H21A	108.7	H49A—C49—H49B	107.8
C20—C21—H21A	108.7	C49—C50—C51	113.19 (17)
C22—C21—H21B	108.7	C49—C50—H50A	108.9
C20—C21—H21B	108.7	C51—C50—H50A	108.9
H21A—C21—H21B	107.6	C49—C50—H50B	108.9
C21—C22—C23	112.6 (3)	C51—C50—H50B	108.9
C21—C22—H22A	109.1	H50A—C50—H50B	107.8
C23—C22—H22A	109.1	C50—C51—H51A	109.5
C21—C22—H22B	109.1	C50—C51—H51B	109.5
C23—C22—H22B	109.1	H51A—C51—H51B	109.5
H22A—C22—H22B	107.8	C50—C51—H51C	109.5
C22—C23—H23A	109.5	H51A—C51—H51C	109.5
C22—C23—H23B	109.5	H51B—C51—H51C	109.5
H23A—C23—H23B	109.5	C53—C52—C42	115.35 (12)
C22—C23—H23C	109.5	C53—C52—H52A	108.4
H23A—C23—H23C	109.5	C42—C52—H52A	108.4
H23B—C23—H23C	109.5	C53—C52—H52B	108.4
C25—C24—C14	115.33 (13)	C42—C52—H52B	108.4
C25—C24—H24A	108.4	H52A—C52—H52B	107.5
C14—C24—H24A	108.4	C54—C53—C52	114.01 (13)
C25—C24—H24B	108.4	C54—C53—H53A	108.7
C14—C24—H24B	108.4	C52—C53—H53A	108.7
H24A—C24—H24B	107.5	C54—C53—H53B	108.7
C26—C25—C24	114.49 (14)	C52—C53—H53B	108.7
C26—C25—H25A	108.6	H53A—C53—H53B	107.6
C24—C25—H25A	108.6	C53—C54—C55	113.92 (16)
C26—C25—H25B	108.6	C53—C54—H54A	108.8
C24—C25—H25B	108.6	C55—C54—H54A	108.8
H25A—C25—H25B	107.6	C53—C54—H54B	108.8
C25—C26—C27	114.59 (17)	C55—C54—H54B	108.8
C25—C26—H26A	108.6	H54A—C54—H54B	107.7
C27—C26—H26A	108.6	C56—C55—C54	113.6 (2)
C25—C26—H26B	108.6	C56—C55—H55A	108.8
C27—C26—H26B	108.6	C54—C55—H55A	108.8
H26A—C26—H26B	107.6	C56—C55—H55B	108.8
C28—C27—C26	113.6 (2)	C54—C55—H55B	108.8
C28—C27—H27A	108.8	H55A—C55—H55B	107.7
C26—C27—H27A	108.8	C55—C56—H56A	109.5
C28—C27—H27B	108.8	C55—C56—H56B	109.5
C26—C27—H27B	108.8	H56A—C56—H56B	109.5
H27A—C27—H27B	107.7	C55—C56—H56C	109.5
C27—C28—H28A	109.5	H56A—C56—H56C	109.5
C27—C28—H28B	109.5	H56B—C56—H56C	109.5
H28A—C28—H28B	109.5	C2—O1—C15	117.02 (11)
C27—C28—H28C	109.5	C6—O2—C16	117.76 (12)
H28A—C28—H28C	109.5	C9—O3—C17	118.75 (13)
H28B—C28—H28C	109.5	C13—O4—C18	118.20 (11)
C30—C29—C34	119.75 (13)	C30—O5—C43	116.80 (11)
C30—C29—H29	120.1	C34—O6—C44	117.70 (12)
C34—C29—H29	120.1	C37—O7—C45	119.00 (12)
O5—C30—C29	122.62 (13)	C41—O8—C46	117.68 (11)
			
C6—C1—C2—O1	179.45 (13)	C31—C32—C33—C34	−1.21 (19)
C6—C1—C2—C3	−1.7 (2)	C31—C32—C33—C35	174.97 (12)
O1—C2—C3—C4	−178.09 (12)	C30—C29—C34—O6	178.80 (12)
C1—C2—C3—C4	3.0 (2)	C30—C29—C34—C33	−0.6 (2)
O1—C2—C3—C14^i^	5.19 (18)	C32—C33—C34—O6	−178.93 (11)
C1—C2—C3—C14^i^	−173.70 (13)	C35—C33—C34—O6	4.70 (18)
C2—C3—C4—C5	−2.1 (2)	C32—C33—C34—C29	0.49 (19)
C14^i^—C3—C4—C5	174.45 (13)	C35—C33—C34—C29	−175.88 (12)
C3—C4—C5—C6	−0.1 (2)	C34—C33—C35—C38	−81.52 (16)
C3—C4—C5—C7	−177.63 (13)	C32—C33—C35—C38	102.39 (15)
C2—C1—C6—O2	−179.54 (13)	C34—C33—C35—C47	150.64 (12)
C2—C1—C6—C5	−0.7 (2)	C32—C33—C35—C47	−25.45 (18)
C4—C5—C6—O2	−179.51 (12)	C41—C36—C37—O7	177.96 (13)
C7—C5—C6—O2	−1.86 (19)	C41—C36—C37—C38	−1.7 (2)
C4—C5—C6—C1	1.5 (2)	O7—C37—C38—C39	−176.61 (12)
C7—C5—C6—C1	179.19 (13)	C36—C37—C38—C39	3.1 (2)
C4—C5—C7—C10	−98.86 (16)	O7—C37—C38—C35	1.04 (19)
C6—C5—C7—C10	83.67 (16)	C36—C37—C38—C35	−179.26 (13)
C4—C5—C7—C19	29.4 (2)	C33—C35—C38—C39	−32.11 (19)
C6—C5—C7—C19	−148.07 (14)	C47—C35—C38—C39	97.51 (15)
C13—C8—C9—O3	−179.32 (13)	C33—C35—C38—C37	150.41 (13)
C13—C8—C9—C10	1.6 (2)	C47—C35—C38—C37	−79.97 (16)
O3—C9—C10—C11	177.45 (13)	C37—C38—C39—C40	−1.3 (2)
C8—C9—C10—C11	−3.4 (2)	C35—C38—C39—C40	−178.77 (13)
O3—C9—C10—C7	−2.0 (2)	C38—C39—C40—C41	−1.9 (2)
C8—C9—C10—C7	177.10 (14)	C38—C39—C40—C42	−179.11 (12)
C5—C7—C10—C11	29.8 (2)	C37—C36—C41—O8	179.39 (13)
C19—C7—C10—C11	−99.88 (16)	C37—C36—C41—C40	−1.6 (2)
C5—C7—C10—C9	−150.77 (13)	C39—C40—C41—O8	−177.60 (12)
C19—C7—C10—C9	79.56 (16)	C42—C40—C41—O8	−0.27 (19)
C9—C10—C11—C12	1.7 (2)	C39—C40—C41—C36	3.3 (2)
C7—C10—C11—C12	−178.89 (13)	C42—C40—C41—C36	−179.34 (13)
C10—C11—C12—C13	1.9 (2)	C39—C40—C42—C31^ii^	−32.87 (18)
C10—C11—C12—C14	178.79 (13)	C41—C40—C42—C31^ii^	149.96 (13)
C9—C8—C13—O4	−178.79 (13)	C39—C40—C42—C52	95.80 (15)
C9—C8—C13—C12	2.1 (2)	C41—C40—C42—C52	−81.38 (15)
C11—C12—C13—O4	177.07 (12)	C33—C35—C47—C48	−60.43 (16)
C14—C12—C13—O4	−0.02 (19)	C38—C35—C47—C48	170.21 (11)
C11—C12—C13—C8	−3.8 (2)	C35—C47—C48—C49	−179.75 (13)
C14—C12—C13—C8	179.12 (13)	C47—C48—C49—C50	−174.79 (14)
C11—C12—C14—C3^i^	32.57 (19)	C48—C49—C50—C51	−177.45 (18)
C13—C12—C14—C3^i^	−150.54 (13)	C31^ii^—C42—C52—C53	−65.20 (16)
C11—C12—C14—C24	−97.35 (16)	C40—C42—C52—C53	166.90 (12)
C13—C12—C14—C24	79.54 (16)	C42—C52—C53—C54	−74.83 (17)
C5—C7—C19—C20	63.96 (17)	C52—C53—C54—C55	−175.36 (14)
C10—C7—C19—C20	−167.15 (13)	C53—C54—C55—C56	−179.55 (18)
C7—C19—C20—C21	175.30 (16)	C1—C2—O1—C15	0.5 (2)
C19—C20—C21—C22	65.6 (3)	C3—C2—O1—C15	−178.42 (13)
C20—C21—C22—C23	167.7 (2)	C1—C6—O2—C16	−20.1 (2)
C12—C14—C24—C25	−162.65 (12)	C5—C6—O2—C16	160.93 (13)
C3^i^—C14—C24—C25	67.83 (17)	C8—C9—O3—C17	17.2 (2)
C14—C24—C25—C26	73.33 (18)	C10—C9—O3—C17	−163.64 (17)
C24—C25—C26—C27	174.15 (15)	C8—C13—O4—C18	8.3 (2)
C25—C26—C27—C28	−178.36 (18)	C12—C13—O4—C18	−172.56 (13)
C34—C29—C30—O5	−178.55 (13)	C29—C30—O5—C43	0.0 (2)
C34—C29—C30—C31	1.3 (2)	C31—C30—O5—C43	−179.86 (13)
O5—C30—C31—C32	177.96 (11)	C29—C34—O6—C44	19.75 (19)
C29—C30—C31—C32	−1.94 (19)	C33—C34—O6—C44	−160.84 (13)
O5—C30—C31—C42^ii^	−4.43 (18)	C36—C37—O7—C45	−8.4 (2)
C29—C30—C31—C42^ii^	175.67 (12)	C38—C37—O7—C45	171.30 (16)
C30—C31—C32—C33	1.92 (19)	C36—C41—O8—C46	−5.6 (2)
C42^ii^—C31—C32—C33	−175.61 (12)	C40—C41—O8—C46	175.33 (13)

Symmetry codes: (i) −*x*+2, −*y*, −*z*; (ii) −*x*+1, −*y*+1, −*z*+1.
